# Proinflammatory effect in whole blood by free soluble bacterial components released from planktonic and biofilm cells

**DOI:** 10.1186/1471-2180-8-206

**Published:** 2008-11-27

**Authors:** Jan Oscarsson, Maribasappa Karched, Bernard Thay, Casey Chen, Sirkka Asikainen

**Affiliations:** 1 Oral Microbiology, Department of Odontology, Umeå University, SE-90187 Umeå, Sweden; 2 Primary Oral Health Care, USC School of Dentistry, University of Southern California, Los Angeles, CA90089-0641, USA; 3 Department of Medical Biochemistry and Microbiology, Uppsala Biomedical Center, Uppsala University, SE-75123 Sweden

## Abstract

**Background:**

*Aggregatibacter actinomycetemcomitans *is an oral bacterium associated with aggressive forms of periodontitis. Increasing evidence points to a link between periodontitis and cardiovascular diseases, however, the underlying mechanisms are poorly understood. This study investigated the pathogenic potential of free-soluble surface material, released from live planktonic and biofilm *A. actinomycetemcomitans *cells.

**Results:**

By employing an *ex vivo *insert model (filter pore size 20 nm) we demonstrated that the *A. actinomycetemcomitans *strain D7S and its derivatives, in both planktonic and in biofilm life-form, released free-soluble surface material independent of outer membrane vesicles. This material clearly enhanced the production of several proinflammatory cytokines (IL-1β, TNF-α, IL-6, IL-8, MIP-1β) in human whole blood, as evidenced by using a cytokine antibody array and dissociation-enhanced-lanthanide-fluorescent-immunoassay. In agreement with this, quantitative real-time PCR indicated a concomitant increase in transcription of each of these cytokine genes. Experiments in which the LPS activity was blocked with polymyxin B showed that the stimulatory effect was only partly LPS-dependent, suggesting the involvement of additional free-soluble factors. Consistent with this, MALDI-TOF-MS and immunoblotting revealed release of GroEL-like protein in free-soluble form. Conversely, the immunomodulatory toxins, cytolethal distending toxin and leukotoxin, and peptidoglycan-associated lipoprotein, appeared to be less important, as evidenced by studying strain D7S *cdt*/*ltx *double, and *pal *single mutants. In addition to *A. actinomycetemcomitans *a non-oral species, *Escherichia coli *strain IHE3034, tested in the same *ex vivo *model also released free-soluble surface material with proinflammatory activity.

**Conclusion:**

*A. actinomycetemcomitans*, grown in biofilm and planktonic form, releases free-soluble surface material independent of outer membrane vesicles, which induces proinflammatory responses in human whole blood. Our findings therefore suggest that release of surface components from live bacterial cells could constitute a mechanism for systemic stimulation and be of particular importance in chronic localized infections, such as periodontitis.

## Background

Periodontitis is one of the most common chronic infections in humans, in which overgrowth of subgingival Gram-negative bacteria leads to chronic inflammation and gradual degradation of tooth-supporting tissues. The Gram-negative bacterium *Aggregatibacter (Actinobacillus) actinomycetemcomitans *is implicated in aggressive forms of periodontitis [1,2]. The oral cavity is its natural habitat, but the bacterium can also translocate from the oral cavity into the blood circulation, as evidenced by the occurrence of severe non-oral *A. actinomycetemcomitans *infections [3].

Increasing evidence points to a link between periodontitis and cardiovascular diseases [4-7]. However, the pathogenic mechanisms that would render periodontitis patients to increased cardiovascular risk are still poorly understood. Previous experimental studies on the background of the association between periodontitis and cardiovascular diseases have mainly worked on the basis of the infection hypothesis that suggests that chronic low-grade bacterial and/or viral infections have a causal role in the development of atherosclerosis and its sequels, such as myocardial infarction and stroke [8,9]. It is believed that infections raise systemic inflammatory status, as evidenced by elevated circulating levels of proinflammatory cytokines and acute phase reactants, which in turn may promote endothelial dysfunction and proatherogenic and proinflammatory phenomena in arterial walls [10,11].

Living bacteria can extend their pathogenicity by active extracellular release of surface components. A major route for the release of outer membrane components from Gram-negative bacteria is via shedding of outer membrane vesicles (OMV), which also allow the delivery of pathogenic effector proteins to eukaryotic target cells [12,13]. In addition, secretion of free-soluble outer membrane proteins (OMP) from bacterial cultures of e.g. *Acinetobacter radioresistens *and *Escherichia coli *could be suggested from previous studies [14-16], although the dependence of vesicles was not elucidated. Recently, we addressed the question whether live periodontal pathogens release free-soluble surface components, which could serve as an additional mechanism for spreading bacterial material from periodontal pockets to blood circulation. Interestingly, our results from an *in vitro *insert model, designed to control for bacterial viability and OMV, demonstrated release of peptidoglycan-associated lipoprotein (PAL) and lipopolysaccharide (LPS) in addition to unidentified material from live planktonic *A. actinomycetemcomitans *cells, independent of OMV [17].

In periodontal pockets, bacteria grow on tooth surfaces as biofilms. Whether the biofilm bacteria also have the capability to release free-soluble surface material to the surrounding environment is not known. As *A. actinomycetemcomitans *PAL in purified form provoked proinflammatory responses in human whole blood *ex vivo *[17] we hypothesize that the extracellular release of free-soluble surface material from live *A. actinomycetemcomitans *cells could constitute a novel pathogenic mechanism that may be of particular importance in chronic localized infections, such as periodontitis. The present study was undertaken as, except for secretion via specialized secretory systems [18], there is limited knowledge of the proinflammatory effects of free-soluble surface material released from live Gram-negative bacteria. Our aim was to investigate in an *ex vivo *model the pathogenic potential of the pool of components released in free-soluble form by live planktonic and biofilm *A. actinomycetemcomitans *cells, and to make an attempt to delineate the identity of the secreted components.

## Results

### Free-soluble material released by *A. actinomycetemcomitans *induces proinflammatory responses in whole blood

To study the pathogenic potential of free-soluble material released by *A. actinomycetemcomitans *cells, we implemented our cell culture plate insert model for stimulation of human whole blood as described in Methods. Similarly to our previous studies using the insert model [17], the absence of *A. actinomycetemcomitans *cell lysis in the wells during the experiments was confirmed by immunoblot analysis of samples taken from the wells outside the inserts (filtrates), using an antiserum raised against the cytoplasmic protein cyclic AMP receptor protein (CRP) (data not shown). In addition, plating revealed no apparent death of *A. actinomycetemcomitans *cells, or contamination in the wells (data not shown).

**Figure 1:**
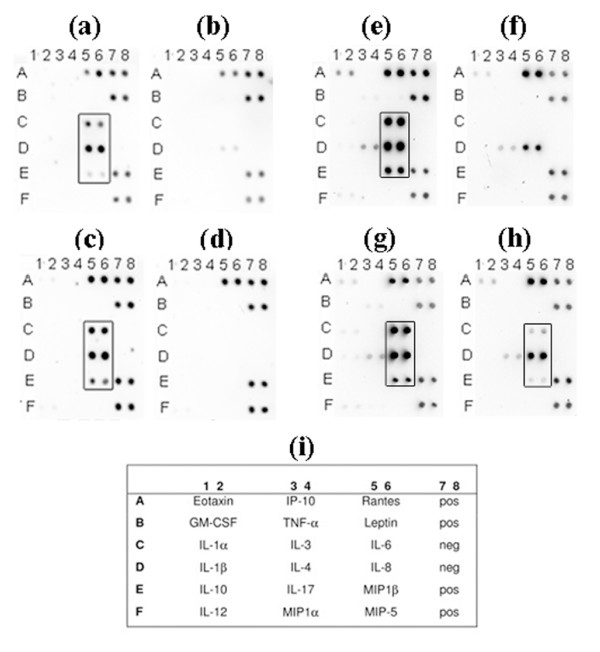
**Cytokine induction in human whole blood by free-soluble material released by live *A. actinomycetemcomitans *cells**. A cytokine antibody array was used to detect cytokines produced by human whole blood after stimulation for 6 h with free-soluble material released from *A. actinomycetemcomitans *as follows: planktonic D7S (panel a), and a corresponding negative control (panel b); planktonic D7SS (panel c), and a corresponding negative control (panel d); biofilm D7S (panel e), and a corresponding negative control (panel f); planktonic D7S Δ*cdt*/*ltx *in the absence (panel g) and in the presence (panel h) of polymyxin B (PMB). Serum (50% in PBS) containing no bacteria served as negative controls. The locations of cytokine antibodies and positive (pos) and negative (neg) controls on the array are indicated (panel i).

According to our findings from the cytokine antibody array analysis, used to screen for the cytokine responses in human whole blood, the free-soluble material released by strain D7S (rough-colony wild type), grown in planktonic form, caused clearly enhanced production of the interleukins IL-6 and IL-8, and of macrophage inflammatory protein (MIP-1β), relative to no bacteria controls (Fig. 1A–B). The same was observed when whole human blood was stimulated with free-soluble material released from *A. actinomycetemcomitans *strain D7SS (smooth-colony variant) (Fig. 1C–D). In addition, very similar results were obtained when whole blood from another donor was used (data not shown), indicating no apparent inter-individual differences. Interestingly, production of IL-6, IL-8 and MIP-1β was also induced when D7S cells grown in biofilm were used (Fig. 1E–F). This suggests that *A. actinomycetemcomitans *cell populations growing in biofilms also release free-soluble material with proinflammatory activity to the surrounding environment. Taken together, we concluded that the free-soluble components released by live *A. actinomycetemcomitans *cells, grown either planktonic or in biofilm, could indeed induce proinflammatory effects in whole blood.

### Free-soluble material released by *A. actinomycetemcomitans *stimulates TNF-α and IL-1β production in whole blood

**Figure 2:**
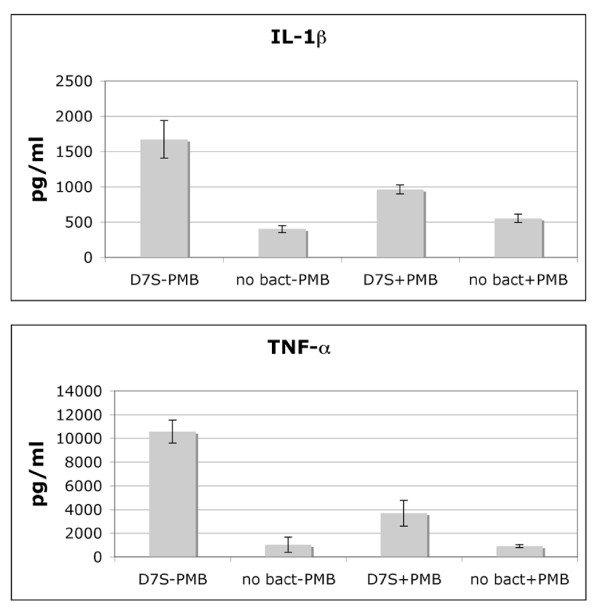
**Induction of IL-1β and TNF-α production in human whole blood by free-soluble material released by live *A. actinomycetemcomitans *cells**. DELFIA was used to quantify the levels of IL-1β and TNF-α in human whole blood after stimulation with planktonic *A. actinomycetemcomitans *strain D7S for 6 h. Serum (50% in PBS) containing no bacteria served as negative controls. The stimulation was done in the presence (+PMB) and in the absence (-PMB) of polymyxin B. Shown are the means and standard deviations from three independent experiments (P < 0.05).

According to the above-described cytokine analyses (Fig. 1) the free-soluble material released from the test strains appeared to have no effect on the production of TNF-α and IL-1β. As a previous report indicated increased production of these cytokines in human whole blood, stimulated with *A. actinomycetemcomitans *LPS [19], the levels of TNF-α and IL-1β were also quantified using a highly sensitive approach (Dissociation enhanced lanthanide fluorescence immuno assay; DELFIA [see Methods]). As shown in Fig. 2, a significant increase in both TNF-α (10.3-fold) and IL-1β (4.2-fold) production was detected when whole human blood was stimulated with free-soluble material released from planktonic D7S cells in the cell culture model (see Methods), relative to no bacteria controls. Why this was not seen using the cytokine array (Fig. 1) is unclear. According to our findings (Fig. 2), the increase in TNF-α and IL-1β production was less pronounced in the presence of polymyxin B (PMB), which blocks LPS activity [20]. However, there was still a clear upregulation (4.1-fold and 1.7-fold, respectively), indicative of an LPS-independent effect on TNF-α and IL-1β production. Thus, we concluded that in addition to IL-6, IL-8 and MIP-1β, the production of TNF-α and IL-1β in whole blood was also enhanced by the free-soluble material released by D7S.

### Free-soluble material released by *A. actinomycetemcomitans *enhances cytokine gene transcription in whole blood

**Figure 3:**
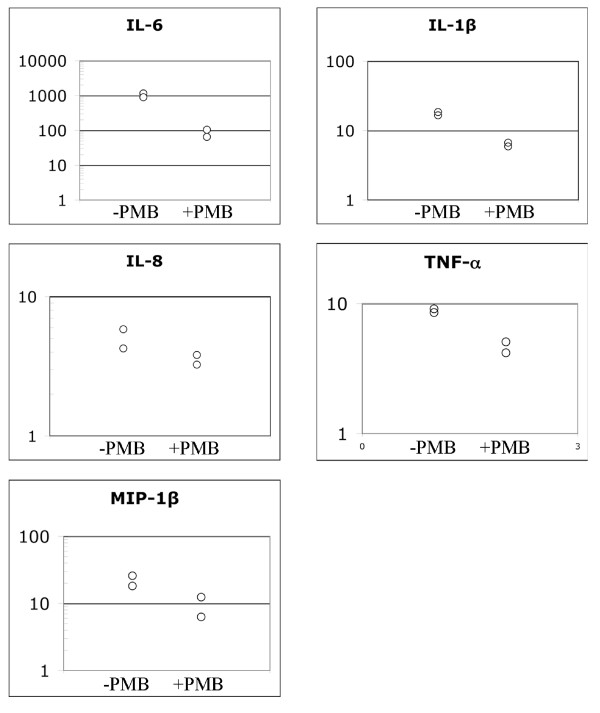
**Induction of cytokine gene transcription in human whole blood by free-soluble material released by live *A. actinomycetemcomitans *cells**. qRT-PCR was used to quantify the levels of IL-1β, IL-6, IL-8, MIP-1β, and TNF-α mRNA in human whole blood after stimulation with planktonic *A. actinomycetemcomitans *strain D7S for 6 h. The stimulation was done in the presence (+PMB) and in the absence (-PMB) of polymyxin B. Shown is the increase (fold change) of each cytokine mRNA in whole blood stimulated with strain D7S relative to no bacteria controls (50% serum in PBS) from two separate experiments (P < 0.05).

To confirm our observations of increased production of the above proinflammatory cytokines and chemokines in stimulated whole blood (Fig. 1 and Fig. 2, the amount of transcript of each gene was quantified using quantitative real-time PCR (qRT-PCR). As indicated in Fig. 3, there was up to 1000-fold increased transcription of each cytokine gene when human blood was stimulated with free-soluble material released from planktonic D7S cells relative to no bacteria controls. Although the increase in cytokine gene transcription was less intensive (up to 100-fold) when the whole blood stimulation was done in the presence of PMB (Fig. 3), each gene was still clearly upregulated, indicating that this effect was also LPS-independent. We could therefore conclude that transcription of IL-6, IL-8, MIP-1β, TNF-α and IL-1β in whole blood was enhanced by the free-soluble material released by D7S.

### LPS-independent cytokine stimulation in whole blood: possible involvement of additional factors released in free-soluble form

**Figure 4:**
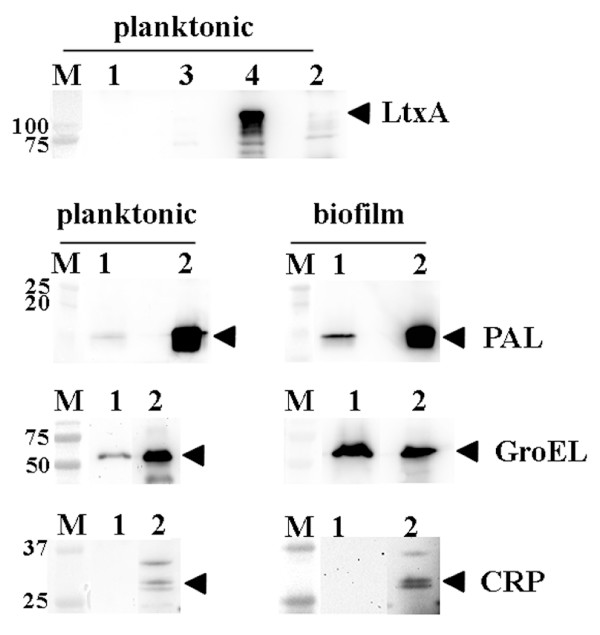
**Immunoblot detection of proteins released in free-soluble form by biofilm and planktonic *A. actinomycetemcomitans *strains, using polyclonal antisera raised against LtxA, PAL, GroEL, and CRP (lysis marker), respectively**. Proteins released from strain D7S to PBS through the 20 nm filters of the inserts were collected after 6 h of incubation and a concentrated sample (denoted 1) equal to 0.75 ml filtrate was applied on the gels where indicated. The following whole cell preparation samples (10 μg protein each) were loaded as controls where indicated: 2. D7S, 3. D7S Δ*cdtABC*/*ltxA*, and 4. JP2 (high producer of LtxA [56]). The sizes of the proteins (kDa) in the prestained molecular weight marker (M) are indicated. The reactive band corresponding to each protein is indicated with an arrow.

As the free-soluble material released by strain D7S provoked proinflammatory responses in human whole blood also independently of LPS, we investigated the possibility that this was due to release of leukotoxin A (LtxA) and/or cytolethal distending toxin (CDT). These immunomodulatory toxins were earlier shown to be extracellularly secreted by *A. actinomycetemcomitans *[21,22]. To test the contribution of LtxA and CDT we constructed a derivative of strain D7S having both the *ltxA *and *cdtABC *gene loci deleted (see Methods). The abolished production of LtxA was confirmed by immunoblotting, using specific antibodies (Fig. 4). According to our findings using the *ex vivo *insert model for stimulation of whole blood, inactivation of *cdtABC *and *ltxA *had no major effect. Instead, the free-soluble material released by the D7S double mutant, grown in planktonic form, induced a proinflammatory response in whole blood similar to that of the parental strain, D7S (Fig. 1A–B), i.e. production of IL-6, IL-8, and MIP-1β was clearly enhanced (Fig. 1G) relative to no bacteria controls (Fig. 1F). When this experiment was carried out in the presence of PMB (Fig. 1H) to inhibit LPS activity, the production of these cytokines was still induced relative to no bacterial controls (Fig. 1F), again indicating LPS-independent stimulation. However, the cytokine stimulation was less intensive, compared to when PMB was absent (Fig. 1G). Lack of contribution of LtxA and CDT to cytokine stimulation in human whole blood is consistent with the absence of these toxins in the filtrates released by strain D7S to PBS, which was also confirmed by immunoblotting (Fig. 4 and data not shown).

As PAL was identified in the free-soluble material released by both D7S and D7SS [17](Fig. 4), a *pal *mutant derivative (D7SS-p) was subsequently tested in the *ex vivo *model. According to our results, the free-soluble material released by planktonic D7SS-p induced a cytokine response in whole blood very similar to that of the parental strain, D7SS (data not shown). These findings together suggest that other factor(s) released in free-soluble form by *A. actinomycetemcomitans *may be involved in the induction of proinflammatory responses in whole blood.

### GroEL-like protein is a major protein released in free-soluble form by *A. actinomycetemcomitans *D7S

**Figure 5:**
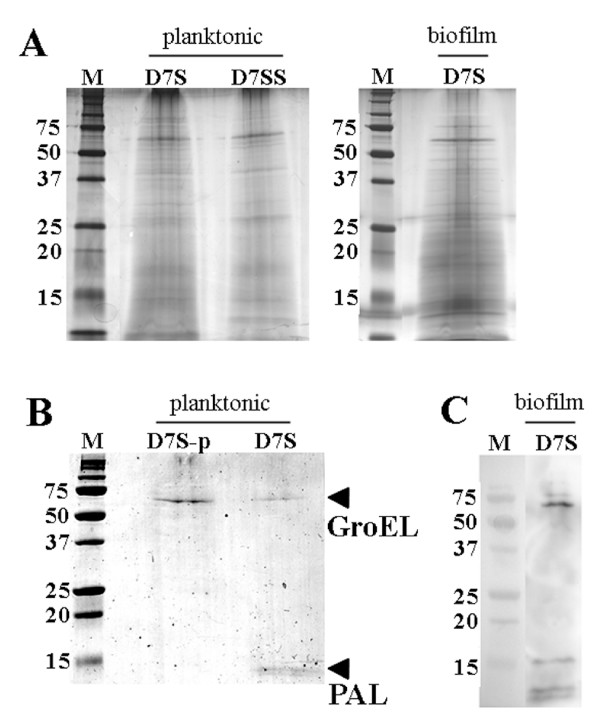
**SDS-PAGE analysis of proteins released in free-soluble form by biofilm and planktonic *A. actinomycetemcomitans *D7S, its isogenic *pal *mutant (D7S-p), or D7SS, detected by Silver-staining (panel A), by Coomassie blue-staining (panel B), and by immunoblotting using an antiserum raised against whole cell antigen of *A. actinomycetemcomitans *serotype a (panel C)**. Proteins released to PBS through the 20 nm filters of the inserts were collected after 6 h of incubation. Concentrated samples equal to 1.5 ml filtrate were applied in each well for Silver- and Coomassie blue-staining, and equal to 0.75 ml for immunoblotting. Protein bands corresponding to GroEL and PAL are indicated with arrows. The sizes of the proteins (kDa) in the prestained molecular weight marker (M) are indicated.

To obtain more information about the free-soluble proteins secreted by *A. actinomycetemcomitans*, we used the cell culture insert model with PBS instead of serum/whole blood (see Methods). Plating revealed no apparent death of *A. actinomycetemcomitans *cells, or contamination in the wells (data not shown). As in our previous studies using the insert model [17], the absence of *A. actinomycetemcomitans *cell lysis in the wells during the experiments was confirmed by immunoblot analysis of filtrates, using an antiserum raised against the cytoplasmic protein, CRP (Fig. 4). SDS-PAGE analysis of filtrates from D7S and D7SS, grown both planktonic and in biofilm form (Fig. 5A), revealed several bands after Silver staining. Finding similar band profiles of material released through the 20 nm pores of the inserts from both D7S and D7SS is in accordance with the cytokine induction by these strains as determined by the present antibody array (Fig. 1).

Since our results hitherto obtained with D7S and D7SS were very similar with each other, we continued our analyses mainly using filtrates from D7S. As shown in Fig. 5B, two major protein bands could be detected in filtrates from D7S, by using Coomassie staining. These bands represented proteins of approximate molecular masses of 60 and 15 kDa, respectively. Bands of these sizes (Fig. 5C and data not shown) were also obtained with immunoblot analysis of the D7S filtrates, using an antiserum raised against whole-cell antigen of *A. actinomycetemcomitans *serotype a. Our previous results show that this antiserum reacts to multiple epitopes in *A. actinomycetemcomitans *[23]. Our current findings (Fig. 5C) therefore suggest that the 60 and 15 kDa-proteins are the two major protein antigens released in free-soluble form under the present conditions. As the 15 kDa-protein band was not detected in filtrates from the *pal*-deficient strain D7S-p (Fig. 5B), we concluded that this protein band represents PAL. This was consistent with immunoblotting (Fig. 4 and data not shown).

To determine the identity of the 60 kDa protein we used MALDI-TOF-MS, which identified *A. actinomycetemcomitans *GroEL-like protein (Hsp60; Cpn60) (Swiss-Prot entry P46398). The release of GroEL-like protein in free-soluble form to PBS by both planktonic and biofilm D7S was confirmed using immunoblotting and an antiserum raised against *E. coli *GroEL (Fig. 4). According to SDS-PAGE (Fig. 5), the *pal*-deficient strain D7S-p released a higher amount of GroEL-like protein than the parental strain. This observation is consistent with the role of the Tol-Pal complex in maintaining outer membrane integrity [24,25]. Taken together, we could conclude that GroEL-like protein is a major protein released in free-soluble form by *A. actinomycetemcomitans *D7S.

### Release of free-soluble material with proinflammatory effect by *E. coli *O18

**Figure 6:**
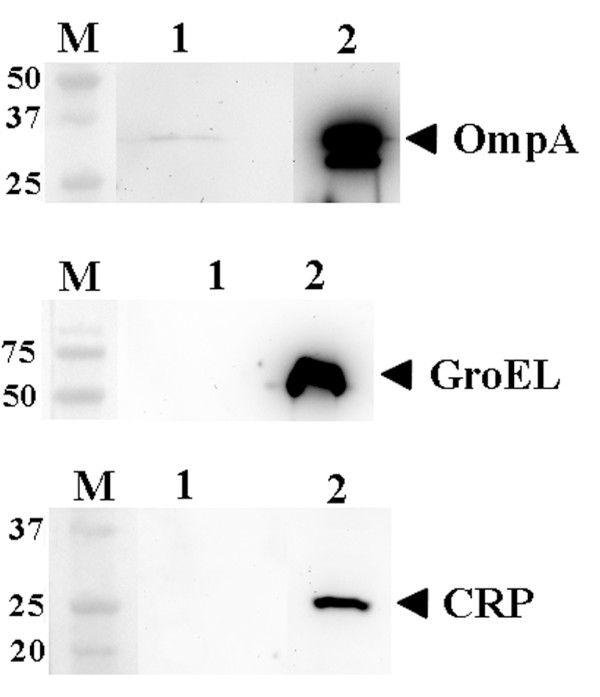
**Immunoblot detection of proteins released in free-soluble form by planktonic *E. coli *strain IHE3034, using polyclonal antisera raised against OmpA, GroEL, and CRP (lysis marker), respectively**. Proteins released to PBS through the 20 nm filters of the inserts were collected after 6 h of incubation and a concentrated sample (denoted 1) equal to 0.75 ml filtrate was applied on the gels where indicated. Whole cell preparation samples (denoted 2; 10 μg protein each) of strain IHE3034 were loaded as controls where indicated. The sizes of the proteins (kDa) in the prestained molecular weight marker (M) are indicated. The reactive band corresponding to each protein is indicated with an arrow.

**Figure 7:**
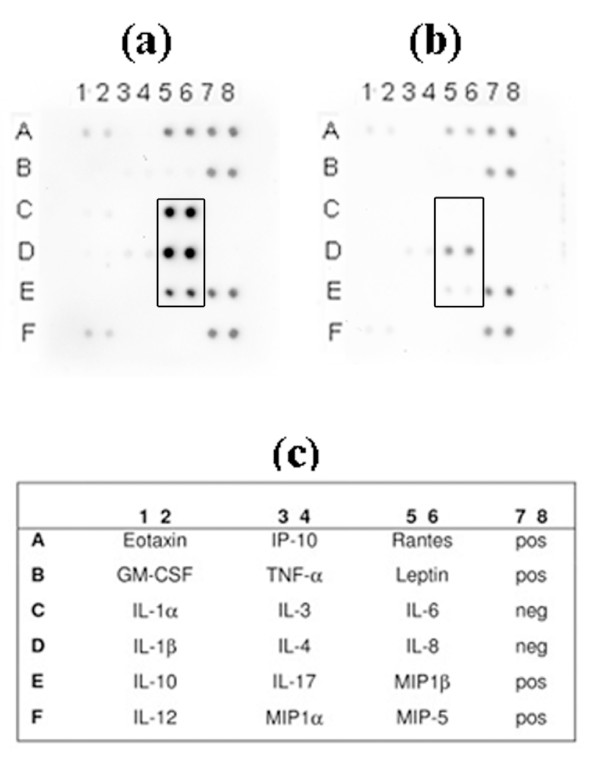
**Cytokine induction in human whole blood by free-soluble material released by live *E. coli***. A cytokine antibody array was used to detect cytokines produced by human whole blood after stimulation for 6 h with free-soluble material released from planktonic *E. coli *IHE3034 (panel a), compared with a corresponding negative control (serum [50% in PBS] containing no bacteria) (panel b). The experiment was carried out in the presence of polymyxin B (PMB). The locations of cytokine antibodies and positive (pos) and negative (neg) controls on the array are indicated (panel c).

To assess the specificity of our present findings to *A. actinomycetemcomitans*, the *ex vivo *culture insert model (Methods), controlled as above (Fig. 6 and data not shown), was used to stimulate human whole blood with free-soluble material released from planktonic *E. coli *strain IHE3034 (serotype O18:K1:H7). As shown in Fig. 7A–C, there was a clear stimulation of IL-6, IL-8, and MIP-1β relative to no bacterial controls, indicative of a proinflammatory effect in whole blood caused by the *E. coli *free-soluble material. This observation is in accordance with previous studies using *E. coli *strains J5 and 789 (serotypes O18:K1:H7 and O78, respectively) [14,15], which demonstrated the release of Outer Membrane Protein A (OmpA), a prime target of the host immune system [26,27]. The release of OmpA in free-soluble form to PBS by planktonic IHE3034 was confirmed using immunoblotting and an antiserum against OmpA (Fig. 6). OMV-independent secretion of OmpA by *E. coli *is consistent with subcellular localization studies, finding OmpA in the fraction of soluble secreted proteins in addition to vesicles [14]. In contrast to OmpA, GroEL was absent from these filtrates (Fig. 6). This is in agreement with the cytoplasmic localization of GroEL and its presence in OMVs released by *E. coli *[28]. Our results therefore show that release of free-soluble surface material with proinflammatory effects in human whole blood is not restricted to *A. actinomycetemcomitans*.

## Discussion

Our present findings using an experimental setup (cell culture insert model) designed to exclude the presence of OMV and bacterial death or lysis in the samples, demonstrated that *A. actinomycetemcomitans *biofilms can release free-soluble surface material. This is consistent with our previous results showing extracellular release of free-soluble surface material (LPS and various proteins) from live planktonic *A. actinomycetemcomitans *cells [17]. This apparently novel, yet uncharacterized mechanism to release soluble material from biofilm to the surrounding environment could be of significance in periodontal pockets, where the biofilm life-form provides persistent bacterial colonization on tooth surfaces. Our present results on *E. coli *show (Fig. 6, 7), however, that release of free-soluble surface material is not restricted to *A. actinomycetemcomitans*, but may differ between bacteria.

By employing the cell culture insert model, we also showed that the released free-soluble material not only from planktonic but also from biofilm *A. actinomycetemcomitans *promoted proinflammatory responses in blood, i.e. IL-1β, IL-6, IL-8, MIP-1β and TNF-α (Fig. 1, 2, 3). This is in agreement with our previous results showing that in addition to elevated serum antibody response to periodontal pathogens in periodontitis patients [29,30], purified *A. actinomycetemcomitans *components induced proatherogenic (foam cells) and/or proinflammatory (cytokines) responses from murine macrophages [31] as well as production of proinflammatory cytokines and chemokines (IL-6, IL-8, and MIP-1β) from human whole blood [17].

The number of live bacteria (1–4 × 10^8^/ml serum) in the present *ex vivo *model exceeded the expected blood concentrations of cultivable bacteria in chronic infections. In serious acute infections the circulating bacterial concentrations appear to be substantially higher by DNA-based than by culture methods as exemplified by a study on meningitis: *Neisseria meningitidis *genome copy density in blood samples ranged from 10^4 ^to 10^8^/ml in patients' blood, although it is likely that these figures also contained dead bacteria [32]. On the other hand, in biofilms bacteria can grow in numbers comparable to our test inoculum [33] and they may release free-soluble material to surrounding tissues or circulation on long-term basis.

Although a substantial stimulatory effect on the production of proinflammatory cytokines in blood was due to LPS, there also was a marked LPS-independent stimulation, which prompted us to also survey the contents of the released free-soluble material. *A. actinomycetemcomitans *has previously been shown to extracellularly secrete two toxins with immunomodulatory activity, leukotoxin A (LtxA) and cytolethal distending toxin (CDT) [21,22]. To assess the contribution of these toxins to cytokine stimulation in whole blood we constructed and tested a novel *ltxA cdtABC *double-deletion mutant derived from a clinical isolate, *A. actinomycetemcomitans *strain D7S. However, as the free-soluble material released from the double mutant had a stimulatory activity, very similar to that secreted from the wildtype strain (Fig. 1), we concluded that released components other than these toxins are likely to be of greater significance. This observation is consistent with our present immunoblot analyses suggesting the absence of LtxA and CDT from D7S filtrates (released to PBS). The lack of LtxA in the filtrates could be a combined result of low levels of leukotoxin produced by D7S under the experimental conditions used (Fig. 4), and of the association of leukotoxin with OMV [34]. It is possible that this could be the case also for CDT, analogous with its association with OMV in *E. coli *[28]. In line with our previous findings using planktonic *A. actinomycetemcomitans *[17], the outer membrane lipoprotein, PAL, was also released in free-soluble form from biofilms (Fig. 4). However, similarly to LtxA and CDT, we did not see a contribution of PAL to cytokine stimulation by using cytokine antibody arrays, *i.e*. inactivation of *pal *had no apparent effect in this experimental setup. This is in contrast to our previous findings that purified PAL stimulated proinflammatory cytokines in whole blood [17]. This discrepancy from the present whole-cell experiment might be a result of enhanced release of LPS in the *pal *deficient mutant strain [17].

In addition to having a crucial role in protein-folding, vital for cell survival during stress [35,36], GroEL (Hsp60; Cpn60)-like proteins from several bacteria, including *A. actinomycetemcomitans*, can activate a plethora of mammalian cells, including macrophages, keratinocytes and periodontal ligament epithelial cells [37-41]. Interestingly, our MALDI-TOF-MS and immunoblot results (Fig. 4, 5) revealed that both bacterial phenotypes, biofilm-form and planktonic *A. actinomycetemcomitans*, released significant amounts of GroEL-like protein in free-soluble form. This observation is consistent with previous studies in which GroEL-like protein was localized to the surface of *A. actinomycetemcomitans *[37,42], as in several other bacteria, e.g. *Borrelia burgdorferi*, *Helicobacter pylori*, *Haemophilus ducreyi*, and *Legionella pneumophila *[43-48]. This supports the previously postulated hypothesis that bacterial GroEL-like proteins may act as direct cell-to-cell virulence factors for host cells [49], albeit the release mechanism is not yet understood. Furthermore, due to the molecular mimicry, the immune response to bacterial GroEL could crossreact with human Hsp60 expressed on endothelial cells, leading to inflammatory reactions. This is supported by finding bacterial GroEL within atherosclerotic lesions in close association with activated inflammatory cells [50], adding further evidence to the involvement of persistent infections such as periodontitis, with atherosclerosis. However, the specific contribution of GroEL to the proinflammatory responses induced in human whole blood would be difficult to assess as this protein is essential for bacterial growth [51]. It also appears to copurify with LPS, suggesting a physical association between these two molecules [52,53]. Analogous to this, it was recently demonstrated that human Hsp60 bound bacterial LPS and synergistically enhanced LPS-induced innate and adaptive immune responses [54]. Thus, the exact nature of the released material that lead to the LPS-independent proinflammatory effects in whole blood is yet to be identified.

Taken together, our present data support that, if entered into the blood circulation, the free-soluble material released from *A. actinomycetemcomitans *has potential to induce proinflammatory responses that are considered important in atherogenesis and used as biomarkers of an elevated risk of cardiovascular events [55]. Within the limitations of our single-species biofilm experiments, we suggest that the release of free-soluble bacterial material from live subgingival biofilms may be a crucial mechanism how chronic inflammation in tooth-supporting tissues is perpetuated and systemic dissemination and immunostimulation are sustained. After entering the parenteral space through the ulcerated periodontal pocket epithelium, free-soluble material may readily gain access into the abundant blood/lymph vascular network immediately under the epithelium, which then opens the route for systemic spread. The hypothesis of the release of free-soluble surface material by live biofilm bacteria, independent of OMV, is new, and although not restricted to oral microorganisms (Fig. 6, 7), it may help extending the knowledge of mechanisms for the host's exposure to pathogenic material originating from the bacterial biofilm in this unique nonparenteral ecological habitat. The next step involves testing the hypothesis in *in vivo *models due to the obvious restrictions in simulating responses to circulating bacterial material by using blood without knowledge of other immunoinflammatory responses of the challenged host.

## Conclusion

Our present study demonstrates that *A. actinomycetemcomitans*, grown in biofilm and planktonic form, releases free-soluble surface material independent of outer membrane vesicles, and that this material induces proinflammatory responses in human whole blood. Our findings therefore suggest that release of surface components from live bacterial cells could constitute a mechanism for systemic stimulation and be of particular importance in chronic localized infections, such as periodontitis.

## Methods

### Bacterial strains and culturing conditions

*A. actinomycetemcomitans *serotype a strains D7S (rough colony type) and D7SS (smooth colony type), their corresponding PAL-deficient mutants, D7SS-p [23] and D7S-p [17], strain D7S Δ*ltx/cdt *(this work), and the leukotoxin highly producing strain, JP2 [56], were all cultured on blood agar plates (5% defibrinated horse blood, 5 mg hemin/l, 10 mg Vitamin K/l, Columbia agar base) incubated in air supplemented with 5% CO_2_, at 37°C for 3 d as previously described [17]. *Escherichia coli *IHE3034 (serotype O18:K1:H7) isolated from meningitis [57] was cultivated 16–18 h on blood agar as described above. For biofilm growth, 2 × 10^8 ^bacterial cells were inoculated in 2 ml tryptic soy broth (Difco) in 24-well cell culture plates (Nunc), which were incubated in static culture in air supplemented with 5% CO_2_, at 37°C for 3 d. Biofilms were stained with crystal violet as previously described [58] and the amount of bound dye, which is proportional to the biofilm mass was quantitated by measuring its absorbance at 590 nm.

### Construction of markerless mutations

The D7S *ltxA cdtABC *markerless double-deletion mutant was constructed using the Cre/*loxP *recombination system, as described previously [59]. In brief, two DNA fragments flanking the target deletion site were generated by PCR and cloned into upstream and downstream *loxP *sites of the vector pLox2-Spe [59], which carries a Spe-resistance (Spe^R^) cassette flanked by *loxP *sites. The recombinant plasmid was subsequently introduced into strain D7S, using natural transformation [60], selecting for spectinomycin-resistant transformants. The replacement of the target gene with *loxP*-Spe cassette-*loxP *was confirmed by PCR sequencing. The Spe cassette in the transformants was then removed by introducing the plasmid pAT-Cre [61], carrying the Cre recombinase. The removal of the Spe cassette was also confirmed by DNA sequence analysis. The resultant mutants had the *cdtABC *operon replaced by a *loxP *site spacer AGATCTGC, and the *ltxA *gene replaced by a *loxP *site with spacer ATGTATAC. This double-deletion strain construct was confirmed by PCR analysis, using the primer pairs CDT1 (5'-GGAGGCGATAACTCTACATCAGG-3') and CDT2 (5'-GTGTCACGTCGTCAAGCCGATG-3'), and LTX1 (5'-CTACTACGGGACCTGTCGCAGG-3') and LTX2 (5'-CCGGCTTTAGTAGCATTACGACCG-3'), respectively.

### Whole blood stimulation

All procedures were conducted in accordance with the guidelines of the local ethics committee at the Medical Faculty of Umeå University, which are in compliance with the Declaration of Helsinki (59^th ^WMA General Assembly, Seoul, October 2008). Blood was drawn from a healthy volunteer, after informed consent, from antecubital vein into blood collection tubes containing heparin (BD Biosciences). A cell culture insert model, described previously [17], was then used to expose the whole blood to free-soluble components released by live *A. actinomycetemcomitans *and *E. coli *cells. For whole blood stimulation using planktonic bacteria, bacterial cells (final concentration 1 × 10^8 ^CFU/ml) were suspended in serum (50% in PBS), separated from the same whole blood. The bacterial suspension (500 μl) was then added to cell culture inserts of pore size 20 nm (Nunc). The inserts were placed into the wells of a 24-well cell culture plate (Nunc), containing 500 μl whole blood. Serum (50% in PBS) containing no bacteria served as a negative control. The cell culture plate was subsequently incubated for 6 h at 37°C in air supplemented with 5% CO_2_.

For whole blood stimulation using biofilm bacteria, *A. actinomycetemcomitans *biofilms (grown 3 d in 24-well cell culture plates; approximately 2 × 10^8 ^CFU) were gently washed with PBS and then 500 μl serum (50% in PBS) was added to the wells. Human blood (500 μl) was then added to cell culture inserts of pore size 20 nm (Nunc), which were placed into the wells with biofilm. The cell culture plate was subsequently incubated as above. When indicated, experiments were performed with PMB (final concentration: 30 μg/ml), an inhibitor of LPS activity [20], to roughly estimate the extent of LPS-independent cytokine stimulation. Similarly to other authors [62], we did not use heat treatment of samples as this has been shown to reduce the cytokine-inducing activity of LPS [63-65].

For RNA isolation from stimulated whole blood, 100 μl blood was removed from the wells after 6 h of incubation and processed as described below. The remaining volume (400 μl) of stimulated whole blood was centrifuged 5 min at 5000 × g. Supernatants were stored at -80°C until cytokine analysis and profiling (see below).

The cell culture insert model was controlled for viability of the bacteria, absence of bacterial lysis, and the absence of bacterial contamination of the material released through the insert filters (filtrates) as described previously [17]. In brief, aliquots from within and outside the inserts were plated on blood agar plates, which were incubated as above for 3 d. Filtrates were also analyzed with immunoblotting, using an antiserum raised against the cytoplasmic protein cyclic AMP receptor protein (CRP). Biofilm integrity was confirmed by crystal violet staining as described previously [66] and also by plating aliquots taken from above the biofilms on blood agar.

### Analysis of free soluble proteins released by live *A. actinomycetemcomitans *and *E. coli *cells

For this, the cell culture insert model was employed and controlled as described above, however with the exception that PBS was used instead of serum or blood. After 6 h of incubation at 37°C in air supplemented with 5% CO_2_, filtrates were collected and precipitated with acetone (80% final concentration) prior to SDS-PAGE analysis. Selected protein bands after Coomassie blue staining were subject to MALDI-TOF-MS analysis at the Umeå Protein Analysis Facility, Department of Chemistry, Umeå University.

### SDS-PAGE and Western immunoblotting

The procedures employed for SDS-PAGE and immunoblot analysis have been described previously [23]. For immunoblots we used the following polyclonal antibodies raised in rabbits against *A. actinomycetemcomitans *LtxA [67] and PAL [68], *E. coli *GroEL (Sigma-Aldrich), *E. coli *OmpA [69], *H. ducreyi *Cdt [70], *V. cholerae *CRP [71], and against whole cells of *A. actinomycetemcomitans *serotype a [72]. Antisera were used at final dilutions of 1:1000, except for the antisera raised against GroEL (1:8000), PAL (1:10000), and OmpA (1:10000). Horseradish peroxidase-conjugated anti-rabbit secondary antibodies were used at a final dilution of 1:10000. Immunoreactive bands were visualized using SuperSignal^® ^(Pierce, Rockford, IL, USA).

### Quantification of cytokine production

To obtain a profile of the cytokines produced by human whole blood after stimulation with released free-soluble bacterial material, a cytokine antibody array (TranSignal™ Human Cytokine Antibody Array 1.0; Panomics, Redwood City, CA, USA), allowing simultaneous detection of the levels of 18 cytokines, was used according to instructions from the manufacturer. Dissociation enhanced lanthanide fluorescence immuno assay [73] was used for the quantification of the amounts of IL-1β and TNF-α produced by human whole blood. For this, 96-well microtiter plate wells were coated with capture-antibodies against human IL-1β and TNF-α (200 ng/well) and incubated overnight at 4°C with shaking. The wells were washed with DELFIA wash buffer twice and saturated with 1.5% BSA in water for 6 h at RT. The wells were then washed twice with 0.9 M NaCl and stabilized at RT for 15 h. Samples and the standards (recombinant IL-1β and TNF-α, e-Bioscience), each in triplicates, were added to the wells and incubated at RT for 1 h. After washing as above twice, detection antibodies against human IL-1β (Europium-labelled) and TNF-α (Samarium-labelled) were added to the wells (200 ng/well) and incubated at RT for 1 h. The wells were subsequently washed six times as above, and then 200 μl DELFIA enhancement solution was added to each well and the plates were incubated at RT on a shaker for 15 min. Europium and Samarium signals were measured using a multilabel reader (Victor^3^, Perkin Elmer).

### Quantitative real-time PCR analysis

Total RNA from whole blood was isolated from two separate stimulation experiments, using the RiboPure™-Blood kit (Ambion Inc.) according to instructions of the manufacturer. Quantitative real-time PCR was performed using the 7900HT Real-time PCR system (Applied Biosystems). Concentration of RNA was determined by measuring the absorbance at 260 nm. One μg of total RNA was converted to cDNA using Transcriptor First Strand cDNA Synthesis Kit (Roche) according to instructions from the manufacturer. Each qRT-PCR reaction (final volume 5 μl) was run in triplicates, containing 1× SYBR^® ^Green PCR Master Mix (Applied Biosystems), and with the cDNA diluted 100× (1000× in addition to assess PCR efficiency). The sequences and final concentrations of the forward and reverse oligonucleotide primers used in qRT-PCR are indicated in Table 1. The qRT-PCR data were normalized to an endogenous control (β-actin).

**Table 1:** Oligonucleotides used in the qRT-PCR reactions

**mRNA target**	**Oligonucleotide (F: forward; R: reverse)**	**Final concentration (nM)**	**Amplicon length (bp)**	**Source**
*β*-actin	F: 5'-GGATGCAGAAGGAGATCACTG-3'	300	90	[74]
	R: 5'-CGATCCACACGGAGTACTTG-3'	300		
IL-1β	F: 5'-ACAGATGAAGTGCTCCTTCCA-3'	300	73	[74]
	R: 5'-GTCGGAGATTCGTAGCTGGAT-3'	300		
IL-6	F: 5'-ACAGCCACTCACCTCTTCAG-3'	300	120	This work
	R: 5'-GTGCCTCTTTGCTGCTTTCAC-3'	300		
IL-8	F: 5'-GAACTGAGAGTGATTGAGAGTGGA-3'	900	134	[75]
	R: 5'-CTCTTCAAAAACTTCTCCACAACC-3'	300		
MIP-1β	F: 5'-CCAAACCAAAAGAAGCAAGC-3'	900	311	[76]
	R: 5'-AGAAACAGTGACAGTGGACC-3'	300		
TNF-α	F: 5'-CCCAGGGACCTCTCTCTAATC-3'	300	84	[74]
	R: 5'-ATGGGCTACAGGCTTGTCACT-3'	300		

### Statistical analysis

Unless otherwise stated all experiments were repeated three times. Mann-Whitney U test was used to assess the statistical significance of differences between experimental and control treatment of whole blood, observed using DELFIA and qRT-PCR. P-values of less than 0.05 were regarded as statistically significant.

## Authors' contributions

JO was responsible for general experimental design, qRT-PCR setup, research supervision, data analysis and interpretation, plus performed the RNA and cDNA work, and wrote the paper. MK developed the insert whole blood stimulation model and the DELFIA experimental protocols, and carried out some of these experiments. BT did most of the qRT-PCR analyses, several immunoblots, and helped in whole-blood stimulation experiments and release studies. CC constructed the D7S *cdtABC*/*ltxA *mutant. SA conceived the project, was responsible for its overall management and vital in drafting the manuscript.
